# Acknowledgement of Reviewers, 2014

**DOI:** 10.5964/ejop.v11i1.939

**Published:** 2015-02-27

**Authors:** Vlad Glăveanu

**Affiliations:** a EJOP Editor; Aalborg University, Aalborg, Denmark

We are grateful for the reliable and helpful support that we have received from the following colleagues, who provided us with valuable editorial help in the peer review process for *Europe's Journal of Psychology* in 2014:

## Handling Editors

Andrew P. Allen
Cherie Armour
Dan Ispas
Maria Kakarika
Maciej Karwowski
Nicholas A. Kuiper
Izabela Lebuda
Rhian Worth

## Reviewers

Hussain Alkharusi
Andrew P. Allen
Frans Orsted Andersen
Ruggero Andrisano Ruggieri
Gamze Arman Incioglu
Kevin Askew
Savita Bakhshi Anand
Baptiste Barbot
Claude Belanger
Flavia Cangia
Melissa Care
Jan Cieciuch
Carlo Combi
Jennifer Henderlong Corpus
Anna Czarna
Daniel David
Aoife De Brún
Constance de Saint-Laurent
Nicolas Deuschel
Japinder Dhesi
Kamila Dobrenko
Dorota Dziedziewicz
Orlando Espino
Alejandro Jose Estudillo
Diana Fleischman
Krzysztof Fronczyk
Aleksandra Gajda
Fabienne Gfeller

Leigh Gibson
Panagiotis Gkorezis
Susan G. Goldberg
Helena Gonzalez
Alessio Gori
Nobuhiko Goto
Jacek Gralewski
William Peter Hampes
Rebecca Harris
Steven Hertler
Katrijn Houben
Gazi Islam
Myrthe Jacobs
Kathryn H. Jacobsen
Maria Kakarika
Pavlo Kanellakis
Paul Kennedy
Johan Lundin Kleberg
Theano Kokkinaki
Kent C. Kowalski
Burak Koyuncu
Yugal Kumar
Willem Kuyken
Mariola Łaguna
Tiziana Lanciano
András Láng
Izabela Lebuda
Hye-ryeon Lee
Déborah Levitan
Emmanouela Mandalaki
Silvia Mari
Rod Martin
Salvador Mascarenhas
Rona Moss-Morris
Matthias Müller
Christopher Murray
Jeanne Nakamura
Kengatharan Navaneethakrishnan
Belgin Okay-Somerville
Daniela Onaca
Panayiotis Panayides
Elpida Keravnou Papailiou
Jose Perez
Maria Pertl
Michael Pirson
Artur Pokropek
Danielle Polage
Joanna Rajchert
Rosana Reis
Gilbert Roberts
Sergi Rufi
Costanza Scaffidi Abbate
Stefan Schmertz
Yuval Shahar
Jennifer Sheehy-Skeffington
David Silvera
Steven Stagg
Jonathan N. Stea
Joanna Szen-Ziemianska
Grzegorz Szumski
Hissam Tawfik
Tarık Totan
Eirini Tsachouridi
Odin van der Stelt
Harm Veling
Daljinder Ritu Virk
T. Joel Wade
Natalia Wentink Martin
Agnieszka Wolowicz-Ruszkowska
Rhian Worth
Shigeo Yamamura
Anna Zajenkowska
Tomasz Zoltak

